# Expert Recommendations to Strengthen Chikungunya Outbreak Surveillance and Reporting for Traveler Health Protection

**DOI:** 10.4269/ajtmh.26-0117

**Published:** 2026-05-26

**Authors:** Daniel C. Payne, Karoun H. Bagamian, Jeffery Goad, Nathan D. Grubaugh, Michael A. Johansson, Moritz U. G. Kraemer, Eyal Leshem, Francesca F. Norman, Alfonso J. Rodríguez-Morales, Henrik Salje, Stephen Thomas, Scott C. Weaver, Lindsey A. Laytner, Rebecca Sturgis, Suzanne K. Scheele, Davidson H. Hamer

**Affiliations:** ^1^ Division of Infectious Diseases, Cincinnati Children’s Hospital Medical Center and University of Cincinnati College of Medicine, Cincinnati, Ohio, USA;; ^2^ Bagamian Scientific Consulting, LLC, Gainesville, Florida, USA;; ^3^ Department of Environmental and Global Health, University of Florida, Gainesville, Florida, USA;; ^4^ School of Pharmacy, Chapman University, Irvine, California, USA;; ^5^ Department of Epidemiology of Microbial Diseases, Yale School of Public Health, New Haven, Connecticut, USA;; ^6^ Bouvé College of Health Sciences, Northeastern University, Boston, Massachusetts, USA;; ^7^ Pandemic Sciences Institute and Department of Biology, University of Oxford, Oxford, England;; ^8^ Center for Travel Medicine and Tropical Diseases, Sheba Medical Center, Tel Hashomer, Israel;; ^9^ National Referral Unit for Tropical Diseases, Infectious Diseases Department, Hospital Universitario Ramón y Cajal, Ramón y Cajal Institute for Health Research (IRYCIS), Centro de Investigación Biomédica en Red de Enfermedades Infecciosas (CIBERINFEC), Madrid, Spain;; ^10^ Faculty of Health Sciences, Universidad Científica del Sur, Lima, Peru;; ^11^ Grupo de Investigación Biomedicina, Faculty of Medicine, Fundación Universitaria Autónoma de las Américas-Institución Universitaria Visión de las Américas, Pereira, Colombia;; ^12^ Department of Genetics, University of Cambridge, Cambridge, England;; ^13^ Global Health Institute, State University of New York Upstate Medical University, Syracuse, New York, USA;; ^14^ Institute for Human Infections & Immunity, University of Texas Medical Branch, Galveston, Texas, USA;; ^15^ Epidemiology and Vaccine Access, Bavarian Nordic, Durham, North Carolina, USA;; ^16^ Department of Global Health, Boston University School of Public Health, Boston, Massachusetts, USA;; ^17^ Section of Infectious Diseases, Department of Medicine, Boston University Chobanian & Avedisian School of Medicine, Boston, Massachusetts, USA;; ^18^ Center on Emerging Infectious Diseases, Boston University, Boston, Massachusetts, USA;; ^19^ National Emerging Infectious Diseases Laboratory, Boston University, Boston, Massachusetts, USA

## Abstract

Chikungunya, a debilitating mosquito-borne disease caused by chikungunya virus, has surged dramatically in the past two decades. Chikungunya outbreaks can intensify rapidly, with infections posing a significant endemic health burden in tropical/subtropical areas and for travelers. Health professionals require timely and accurate outbreak information to adequately protect travelers and assess chikungunya risk. However, detection is often insufficient in the areas most susceptible to outbreaks, leading to diagnostic and reporting delays. Consequently, travelers are often unaware of increased chikungunya infection risk at their destination. We assessed chikungunya surveillance and reporting resources, and we developed expert recommendations for traveler health protection. Because data-reporting cadences, reliability, and availability vary, we organized structured activities and convened an expert panel to compare these resources and evaluate their strengths and weaknesses. We provide a synthesis of the expert panel discussion and provide recommendations on how to improve chikungunya surveillance and risk communication to travelers. Assessing outbreak risk requires using multiple local and international reporting resources because no single resource contains all of the necessary information. The panelists identified several challenges regarding chikungunya surveillance and outbreak reporting: inadequate chikungunya surveillance infrastructure and diagnostic capacity; varying case definitions; limited understanding of the local health care context; inadequate awareness of chikungunya in health care settings; and restricted or delayed access to real-time surveillance data. As chikungunya risk increases worldwide, greater attention and resources are needed to improve timely, sensitive, and specific diagnostic testing and rapid communication to health care workers and travelers, particularly in resource-poor regions where outbreaks occur.

## INTRODUCTION

Chikungunya, a debilitating mosquito-borne disease caused by chikungunya virus, has surged dramatically in the past two decades. Chikungunya poses a significant endemic disease burden and risk to international travelers visiting areas experiencing outbreaks, which can escalate rapidly. Outbreaks have occurred worldwide, predominantly in tropical and subtropical regions where *Aedes aegypti* mosquitoes are primary transmission vectors. Given the increasingly widespread distribution of another competent *Aedes* vector (*Aedes albopictus*), chikungunya virus has started to exploit new ecological niches, including those in more temperate climates. Infected travelers returning from outbreak regions can introduce chikungunya virus to nonendemic areas,^1^ which may spark local transmission. Autochthonous cases have been increasingly documented in Europe,^2^ and in 2025, the first locally acquired, nontravel-related case was confirmed on the northeastern seaboard of the United States.^3^^,^^4^

Chikungunya is more common and can cause significantly poorer health outcomes than travelers and residents of endemic areas currently appreciate.^5^^,^^6^ The global chikungunya burden has been estimated at 35 million infections and 3,700 deaths annually.^7^^,^^8^ All age groups are at risk of disease; however, females and older adults have an elevated risk of symptomatic disease,^9^ and symptomatic maternal cases have been linked to perinatal complications.^10^ Approximately half of acute chikungunya infections result in chronic arthralgias that impair everyday activities, and one in eight confirmed case-patients reports persistent joint pain lasting at least 40 months after infection.^11^^–^^13^ Fatal cases (approximately one per 1,000 infections) mainly occur in neonates, infants, and older adults.^9^ Chikungunya is associated with an increased mortality risk for up to 84 days after symptom onset.^14^

Currently, there are no chikungunya-specific antiviral treatments. However, two chikungunya vaccines (IXCHIQ^®^, a live-attenuated vaccine [Valneva, Saint-Herblain, France], and VIMKUNYA^®^, a virus-like particle vaccine [Bavarian Nordic, Hellerup, Denmark]) have received regulatory approvals in several countries worldwide.^15^^,^^16^ Gavi, the Vaccine Alliance, has not yet adopted these vaccines for funding in lower-income populations. The European Medicines Agency has authorized both vaccines, whereas the US Food and Drug Administration currently authorizes only the virus-like particle vaccine. Recommendations differ across Europe, but in the United States, the recommendation is for use in individuals 12 years old and older traveling to areas with active chikungunya outbreaks and laboratory workers at risk of exposure to chikungunya virus. In the United States, the recommendation also suggests that the vaccine be considered for individuals 12 years old and older moving to or planning to spend prolonged periods in areas with elevated chikungunya risk.^15^^,^^16^

Epidemiologists, travel medicine clinicians, and public health professionals require swift, accurate, and reliable outbreak reporting to assess risk and protect travelers from chikungunya virus infections. Chikungunya outbreak detection, like that of many pathogens, is often insufficient in the areas most vulnerable to outbreaks, leading to diagnostic delays and reporting lags.^17^^,^^18^ Cross-sectional seroprevalence studies have demonstrated that case-based surveillance systems have completely missed entire epidemics in countries such as the Philippines and Burkina Faso,^19^^,^^20^ and surveillance in endemic areas has also revealed low-level transmission between outbreaks.^21^ Surveillance reports and outbreak information can be delayed even in high-income countries. International awareness of an outbreak typically occurs only after it has spread substantially, often because of limited diagnostic and reporting resources. In the absence of rigorous testing, febrile illness is often reported as dengue in countries where both viruses cocirculate.^22^^–^^24^ These delays can lead to travelers being unaware of increased disease risk at their destination. Indeed, chikungunya outbreaks and hidden ongoing transmission have been identified in sentinel travelers, emphasizing the need to strengthen surveillance in endemic countries.^25^^,^^26^

The overall goal of this report was to identify challenges in chikungunya outbreak surveillance and reporting and to develop expert recommendations to enhance these resources to better protect travelers. We convened an expert panel to compare chikungunya outbreak-reporting and risk assessment tools. Here, we synthesize the expert panel’s insights to achieve the following:

1. examine the strengths and weaknesses of current chikungunya risk assessment resources to identify gaps that need to be addressed;
2. determine what is needed to address these existing surveillance and reporting gaps to improve the timeliness and accuracy of chikungunya surveillance; and
3. recommend ways to improve chikungunya surveillance, risk assessment, and risk communication to travelers.

## MATERIALS AND METHODS

### Overview.

This study consisted of two primary activities: 1) a review and comparison of infectious disease-reporting resources that provide chikungunya outbreak information and 2) an expert panel discussion addressing this topic. These activities received institutional review board approval at Cincinnati Children’s Hospital Medical Center.

### Convening of the expert review panel.

An international panel of chikungunya experts (hereafter, the “expert panel” or “panelists”) was convened virtually in September 2025 to identify and characterize key gaps in chikungunya outbreak surveillance and to determine the optimal characteristics and methods for disseminating risk information to effectively engage the public in taking health precautions. The panel consisted of participants with expertise in epidemiology, infectious disease modeling, travel medicine, vaccinology, vector biology, and virology who are all involved in assessing and communicating chikungunya risk worldwide. The session was recorded and transcribed in English using Microsoft Teams software. Panelists’ perspectives, identified challenges, and recommendations were extracted from the transcript and are presented in the panel discussion results. Their preferred outbreak-reporting resources were also summarized. Given the complexity of the discussion, the primary challenges related to chikungunya risk assessment and the corresponding recommendations were synthesized and organized into thematic categories, with each recommendation numbered for clarity.

### Preparation for the expert panel discussion.

#### Review and comparison of chikungunya outbreak-reporting sources.

Study personnel identified chikungunya outbreak-reporting resources through an internet search for online resources describing chikungunya surveillance and case reporting in English, French, and Spanish. Then, a summary table was compiled to describe and compare publicly available chikungunya outbreak-reporting sources, including their methods, operational contexts, regional coverage, reporting speeds, information depth, and other relevant characteristics.

#### Discussion materials.

Expert panel members’ insights were collected first through a brief presession informational survey that gathered their opinions on the identified chikungunya resources. The expert panelists then received a prepanel document that compared outbreak-reporting resources and included a summary table to prepare them for the panel discussion. The investigators prepared discussion questions and relevant probes *a priori*, and they modified them as needed after receiving the results of the informational survey (Supplemental Table 1).

## RESULTS

### Prepanel activity: Outbreak-reporting resources comparison.

**Figure 1.**
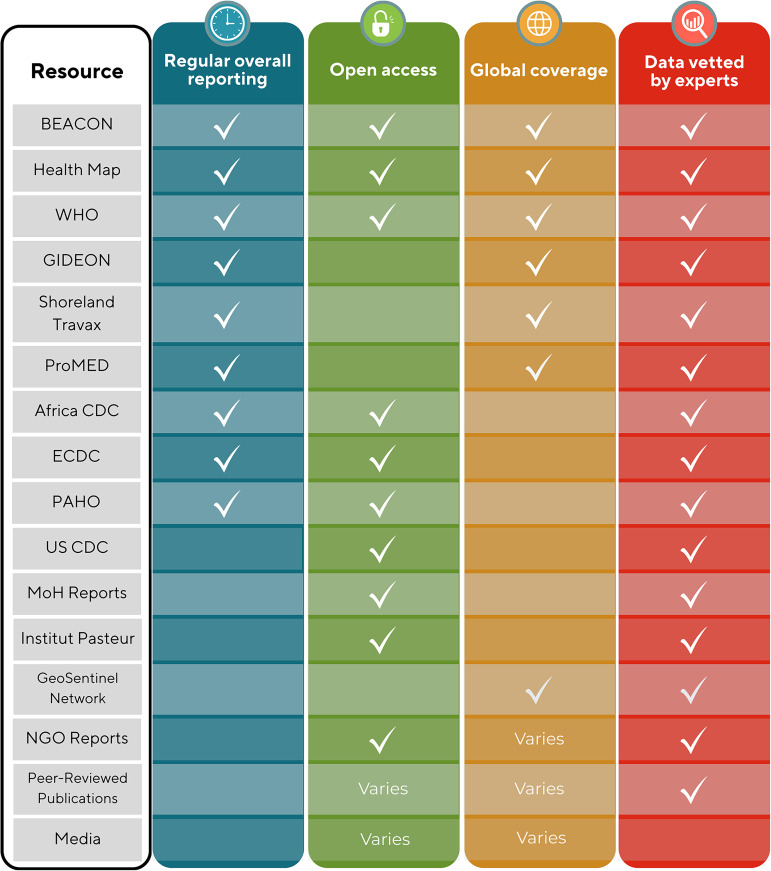
Key outbreak-reporting characteristics of reviewed resources. Africa CDC = Africa Centres for Disease Control and Prevention; BEACON = Biothreats Emergence, Analysis, and Communications Network; ECDC = European Centre for Disease Prevention and Control; GIDEON = Global Infectious Diseases and Epidemiology Online Network; MoH = Ministry of Health; NGO = nongovernmental organization; PAHO = Pan American Health Organization; ProMED = Program for Monitoring Emerging Diseases.

Sixteen online resources were identified as potential outbreak-reporting sources to inform chikungunya risk assessments ranging from governmental and international health-related entities (e.g., WHO, different national or regional CDCs, and Ministries of Health [MoHs]) to expert reporting networks and surveillance programs (e.g., the Biothreats Emergence, Analysis, and Communications Network [BEACON] and HealthMap) to subscription-based services (e.g., the Global Infectious Diseases and Epidemiology Online Network [GIDEON] and Shoreland Travax) (Figure 1). Most of these resources are entirely publicly available (*n* = 12/16) and report having subject matter experts evaluating or verifying the collected and shared data (*n* = 15/16), although the level of expert review varies by resource.

Almost half of the resources (*n* = 7/16) provide global coverage, whereas six resources offer regional or country-specific information, and three have variable coverage (Figure 1). Most global resources have regular reporting cadences, with certain governmental entities (e.g., WHO, the European Centre for Disease Prevention and Control [ECDC], and the Pan American Health Organization [PAHO]) providing weekly updates to their overall disease reporting and nongovernmental networks and tools (such as ProMED [Program for Monitoring Emerging Diseases], HealthMap, BEACON, Shoreland Travax, and GIDEON) updating their data daily. Regarding regional resources, the websites of ECDC, PAHO, and Africa CDC update their overall disease reporting weekly, whereas MoHs, Institut Pasteur, and the US CDC have variable reporting cadences, typically triggered by specific events. Most resources issue situational reports on chikungunya only in response to outbreaks rather than providing continuous updates (Figure 1; Table 1).

**Table 1:** Comparison of key features of infectious disease-reporting resources relevant to chikungunya risk assessment

Resource	Type	Coverage	Overall Update Frequency	Chikungunya Update Frequency	Access	Reported Data
Biothreats Emergence, Analysis, and Communications Network (BEACON)	Open-source surveillance program	Global populations	Daily	Situational	Publicly available	AI-detected emerging biothreat alerts and summaries
HealthMap (Boston Children’s Hospital)	Real-time outbreak surveillance interactive platform	Global populations	Daily	Daily	Publicly available	Location-specific outbreak alerts and maps
WHO	International health agency	Global (all populations in WHO member countries)	Weekly	Situational	Publicly available	Case counts, confirmed cases, outbreaks, risk assessments
Global Infectious Diseases and Epidemiology Online Network (GIDEON)	Commercial surveillance database	Global populations	Daily	Situational	Subscription based	Case counts, confirmed cases, mortality, outbreaks, seasonal trends, geographic spread
Shoreland Travax	Travel medicine decision support tool	Global populations; international travelers	Daily	Situational	Subscription based	Destination-specific risk assessments, outbreaks, safety information, vaccine/prophylaxis recommendations
ProMED (ISID)	Expert-moderated outbreak-reporting system	Global populations	Daily	Situational	Primarily subscription based	Narrative outbreak reports, early warning reports, moderator commentary
Africa Centres for Disease Control and Prevention (Africa CDC)	Regional health agency	All populations in African Union member states	Weekly	Situational	Publicly available	Regional and country-specific outbreaks, geographic spread, country summaries, alerts
European Centre for Disease Prevention and Control (ECDC)	Regional health agency	European Union/European Economic Area residents and international travelers	Weekly	Situational	Publicly available	Local and travel-associated cases, confirmed cases, seasonal trends, geographic spread, outbreaks
Pan American Health Organization (PAHO)	Regional health agency (Americas)	Populations in PAHO member states (North, Central, and South America; the Caribbean)	Weekly	Weekly	Publicly available	Case counts, confirmed cases, epidemiological trends, geographic spread, outbreaks
CDC	National health agency	U.S. residents and international travelers	Variable	Situational	Publicly available	Local and travel-associated case counts, confirmed cases, travel advisories, geographic spread, outbreaks, vaccine recommendations
National, state, or local governmental resources (Ministry of Health [MoH] reports or websites)	National, state, or local health agency	Populations served by the national, state, or local agency	Variable	Situational	Publicly available	Case counts, confirmed cases, public health alerts, outbreak reports, vaccine recommendations
Institut Pasteur	Research institution	Populations served by the Pasteur Network institutes (Africa, Americas, Asia-Pacific, Europe)	Variable	Situational	Publicly available	Outbreak investigations, molecular data, epidemiologic analysis
GeoSentinel Surveillance Network	Clinician-based sentinel traveler and migrant surveillance system	International travelers and migrants	Variable	Situational	Restricted to member clinics	Travel-related disease cases, geographic exposure surveillance
Nongovernmental reports	NGOs, humanitarian efforts, research consortiums	Populations served by the NGOs (global, regional, national, or local)	Variable	Situational	Publicly available	Case counts, outbreaks, alerts, safety reports
Peer-reviewed publications	Academic journals and literature	Study populations	Variable, often delayed	Situational	Publicly available or subscription based	Research data, case reports, outbreak investigations, surveillance studies
Media	News/journalism	Global, regional, national, or local populations affected by reported event	Variable	Situational	Publicly available or subscription	Outbreaks, case counts, alerts
Other	Mixed (social media, word of mouth, unofficial)	Global, regional, national, or local populations affected by reported event	Variable	Variable	Publicly available	Outbreaks, cases, alerts

AI = artificial intelligence; ISID, International Society for Infectious Diseases; NGO = nongovernmental organization; ProMED = Program for Monitoring Emerging Diseases. Hyperlinks are provided for each resource. For national, state, or local governmental resources as well as nongovernmental reports and peer-reviewed publications, we provide a link to an example resource.

Most resources provide multiple types of outbreak-related information. The most commonly reported features across all of the resources were outbreak reports and alerts (*n* = 15/16) followed by the reporting of confirmed cases (*n* = 8/16) (Table 1; Supplemental Figure 1).

### Panel discussion results: Outbreak-reporting resources.

The panelists collectively agreed that they typically draw on multiple resources for chikungunya risk assessments, emphasizing that both local and international resources are crucial for informing them about outbreak risk and characteristics. Their most commonly used and trusted resources for obtaining information about a chikungunya outbreak were publicly available data produced by international, regional, or national governmental entities (e.g., ECDC, WHO, PAHO, and US CDC), which often aggregate locally collected data. ProMED and Shoreland Travax were the most trusted nongovernmental services because subject matter experts vet their data, and they have a rapid reporting turnaround time, critical for capturing an outbreak’s trajectory. Despite reporting delays, peer-reviewed publications produced by infectious disease experts were trusted nongovernmental resources. Some panelists reported using other resources (e.g., media, nongovernmental organization reports, and GIDEON) as supplemental sources for outbreak-reporting information.

Panelists agreed that no single resource was universally suitable across all use scenarios, and their resource preferences depended on their primary occupational objective: travel medicine, vaccinology, public health, or other research. Regardless of their specialization, all panelists agreed that case reports and detailed metadata improve overall risk estimates, but clinical experts favored more accurate real-time assessments for patient care. For clinical or vaccination decisions, timely access to outbreak data was critical—particularly indicators showing whether transmission is emerging, persisting, or waning; however, such data are often difficult to obtain, or their dissemination is often delayed. Detailed information about case transmission rates was not only relevant for consultations on preventive pretravel measures, but it was also required by travel medicine clinicians to enable rapid chikungunya diagnosis and care coordination upon the traveler’s return. For these purposes, preferred resources included Shoreland Travax and GIDEON. Nonclinical researchers, especially those developing chikungunya transmission or predictive risk models, require precise local data, ideally with comprehensive metadata. However, those data are rarely publicly available or machine readable (e.g., some of this information is contained in government reports in PDF files). From vaccine developer and researcher perspectives, obtaining estimates of the baseline transmission levels within an area is paramount. Accordingly, they preferred resources provided by the WHO, ECDC, PAHO, and peer-reviewed publications. These resources also provide information to help identify which countries or regions exhibit consistent reporting and diagnostic practices, thereby gauging their suitability for inclusion in future clinical trials or interventions. In the Supplemental Results, we individually describe the infectious disease-reporting resources highlighted by the panelists. Brief descriptions and hyperlinks for each resource are included in Table 1.

### Panel discussion results: Challenges in chikungunya risk assessment.

#### Principal themes.

The panel explored several challenges regarding chikungunya surveillance and outbreak reporting and how these obstacles affect chikungunya risk assessment under the following five themes: 1) inadequate chikungunya surveillance infrastructure and diagnostic capacity; 2) varying case definitions; 3) limited understanding of the local health care context (e.g., health care-seeking behavior); 4) underappreciation of chikungunya and the scope of its burden in health care settings; and 5) restricted and delayed access to real-time surveillance data.

#### Inadequate chikungunya surveillance infrastructure and diagnostic capabilities: Quality of locally collected data.

*“Ultimately, all data comes from local or national resources. It is hard to go to all sites to find the data, so we use resources that aggregate data. The issue remains that all of these resources still rely on local reporting—they are only as good as the local reporting.” —Panelist 9*

Panelists emphasized the importance of highly accurate and transparent local surveillance data as every outbreak report or risk assessment ultimately relies on local or national epidemiology and response efforts. International resources that aggregate and present data (e.g., WHO and ECDC) depend on data reported to them by each affected country. Most global chikungunya surveillance gaps arise from reduced surveillance and diagnostic capacity in low- to middle-income countries where the disease is more frequently endemic. Even in countries with recurrent arboviral outbreaks, expected robust diagnostic and reporting systems are often missing. Many small, sporadic chikungunya outbreaks go undetected or unreported because of a lack of laboratory diagnostics. These outbreaks can be significant locally and pose risks to travelers.

#### Misdiagnosis and underreporting of chikungunya cases.

*“One of the biggest challenges is trying to gather data on those people who have no laboratory confirmation diagnosis but probably do have chikungunya.” —Panelist 7*

A leading challenge for chikungunya (and other mosquito-borne illnesses) is misdiagnosis or underdiagnosis because of inadequate laboratory diagnostic capacity, knowledge gaps about the disease, and syndromic overlap with other infections. Chikungunya cases, especially milder cases, can be misclassified with other arboviral diseases (e.g., dengue and Zika) and other febrile illnesses, leading to underreporting.^23^ In the absence of laboratory testing, milder chikungunya cases may remain undiagnosed or misdiagnosed as dengue unless the patient develops chronic arthralgia weeks after the acute phase.

Even in endemic countries with adequate diagnostic and reporting systems, coverage gaps persist, and testing criteria remain poorly defined. Routine chikungunya testing is often absent or poorly organized, making it challenging to characterize nonconfirmed cases. Although the CDC and ECDC are considered reliable in reporting confirmed cases, limited testing capacity and chikungunya’s variable clinical presentation impede the accurate estimation of the total number of symptomatic cases. This challenge is exacerbated by asymptomatic infections during an outbreak, where it is seldom achievable to accurately estimate or characterize an outbreak’s true scale based solely on individuals with a laboratory confirmed diagnosis.

#### Inconsistent case definitions.

*“Lots of places use different case definitions, right? So, there are clinically suspect cases, there are epidemiologically suspect cases, and sometimes some of those are called confirmed based on clinical or epi information. That’s not laboratory confirmation. And so across these, there’s also a mix of those definitions that people are using to make it hard to understand what the real underlying burden is, but also how the different locations compare to each other based on what they’re reporting.” —Panelist 1*

Panelists highlighted several issues concerning chikungunya case definitions that obscure an area’s true chikungunya burden and risk, thereby complicating the comparison of burden across different locations. Many of these issues stem from local and regional variation in case definitions.

For diseases that have overlapping signs and symptoms, limited laboratory testing, and outbreak potential, surveillance systems typically use different terminology to convey the level of certainty regarding the etiology of identified cases. The most commonly used terms are suspected (a person shows clinical symptoms consistent with the disease but lacks laboratory confirmation), probable (a person shows suspected disease-consistent symptoms and has an epidemiological link but has inconclusive or unavailable laboratory results), and confirmed case (a person meets clinical criteria and has laboratory confirmation of the disease). First, although outbreak response and reporting entities use chikungunya case definitions, these definitions are not standardized, and probable case definitions for chikungunya are often broad. Some definitions include more stringent criteria than others (e.g., the GeoSentinel case definition). However, relying solely on laboratory-confirmed cases to estimate outbreak magnitude is not sensitive enough as many cases are likely unrecognized and remain untested.

Example: GeoSentinel chikungunya case definition

- Confirmed chikungunya diagnosis defined as a compatible clinical history plus either virus isolation, positive nucleic acid test, or seroconversion/rising titer in paired sera.
- Probable diagnosis defined as a compatible clinical history with a single positive serology result.

#### Limited understanding of local health care context.

*“Each country, each state has different health care seeking behavior trends. That’s typically only something that can be found out about when in conversation with local public health officials or clinicians in country.” —Panelist 2*

Another significant knowledge gap that affects the accurate evaluation of chikungunya risk is the insufficient understanding of the local health care context and its interplay with chikungunya surveillance. Subtle distinctions in local public health operations related to chikungunya are often uncovered only during discussions with local public health officials and health care practitioners. Greater insight into the relative strength of different countries’ surveillance systems is crucial for assessing international risk.

#### Underappreciation of chikungunya and the scope of its burden in health care settings.

*“The problem sometimes is that after the epidemic, with chikungunya, for example … the surveillance is not the same. There [isn’t] the same perception of the risk of the circulation of the virus.” —Panelist 8*

*“Most patients have never heard about chikungunya unless somebody comes back ill and you diagnose them with chikungunya. Just possibly in the context of VFR [visiting friends and relatives] travel … otherwise I think the awareness is generally not very high about chikungunya specifically.” —Panelist 3*

Heightened attention to chikungunya occurs during its explosive outbreaks but wanes afterward, leading to inconsistent funding for surveillance and disease vigilance. The underappreciation of chikungunya in health care settings results from several misconceptions stemming from a lack of knowledge about the disease itself. Insight into chikungunya’s acute clinical impact is blurred by other misdiagnosed diseases, such as dengue, and its chronic effects are underappreciated. In many areas, public health and health care professionals lack a robust understanding of the most recognized chronic chikungunya symptom, arthralgia, because surveillance efforts do not systematically collect data on this symptom. Although this information may exist in patient medical charts in countries that maintain them, these records often lack links to surveillance data, making it challenging to analyze these data and better understand the impact of chronic disease. Emerging evidence regarding neurological symptoms^27^ suggests that improved surveillance can clarify the frequency and severity of these outcomes, thereby better characterizing the longer-term impacts of chikungunya. Given the varied manifestations of chikungunya, patients may present in many different health care settings and be seen by various specialists, including emergency care or primary care providers, rheumatologists, neurologists, and obstetrician–gynecologists.^28^^–^^30^ Some of these clinicians may have limited familiarity with the disease and its clinical features, particularly as there is a lack of high-quality clinical management guidelines.^31^

#### Restricted and delayed access to real-time surveillance data.

*“The type of data we often are after is line list data, but that’s rarely available … when it is available, it’s hugely informative because it often comes with a fair bit of extra data.” —Panelist 10*

*“More importantly, we need … better connections through all these different data sets. Looking at dengue data, when you compare different ways to compile data from a region, sometimes you get very different numbers and maybe not even the trends match up. There’s still quite a bit of work to do [regarding] compiling data sets from across sources.” —Panelist 2*

Many reporting resources offer a diverse array of data; however, these data are frequently reported at levels that do not meet research requirements. Line list data with detailed information for each case are rarely publicly available, likely because of privacy considerations and limited investigative resources. Line list data can be exceptionally informative when available as they often include laboratory confirmation status and other pertinent details about location, timing, symptoms, and host characteristics, such as age, sex, and underlying health conditions. This information is key for developing predictive models and improving risk assessment tools. Certain variables, such as age, are frequently documented but not consistently reported, complicating the interpretation of critical aspects of the outbreak. Chikungunya tends to present with greater severity in infants and older adults who seek medical attention; therefore, understanding the age distribution of patients during an outbreak can help gauge its severity and risk factors for exposure.

Multiple data sources are needed to accurately assess the risk and true underlying burden of an area. Ideally, combining data on the underlying burden, mosquito vectors, viral genotype, and seroprevalence would best indicate chikungunya risk in an area; however, the availability of this type of data is limited for many regions. To enhance burden and risk assessments, it is essential to better connect and integrate various types of data effectively. Also, in emergency outbreak situations or other instances where rapid data sharing is paramount, the quality assurance of collected data is often minimal, and adjustments for data quality variations are rarely made.

As articulated by one expert (Panelist 4), “[Knowing the current disease] incidence in travelers is kind of the Holy Grail of travel medicine.” Although incidence is critical information, it is difficult to obtain because of selection bias in identifying returning travelers and a lack of a denominator to determine the proportionate risk relative to all travelers to an outbreak country. It is essential not only to assess the risk for a traveler visiting countries where chikungunya is endemic but also, to evaluate the risk of disease importation and local transmission to the traveler’s home country, such as has occurred repeatedly during the global re-emergence of chikungunya during the past 20 years.^2^

## DISCUSSION

### Limitations.

Like other qualitative and subject matter expert-based results, this article reflects the learned experiences and observations of recognized experts in the field, but there may be other worthy opinions and contributions that were not considered.

### Recommendations.

The expert panel identified eight overall recommendations to address the identified gaps and improve chikungunya surveillance and risk assessment as well as three recommendations to enhance chikungunya risk communication for travelers.

## CONCLUSION

### Overall recommendations.

- Increasing laboratory confirmation of cases. Regions with the highest risk of chikungunya outbreaks often lack resources for comprehensive and rapid laboratory confirmation. Developing strategies that simultaneously improve the sensitivity of affordable chikungunya detection (i.e., the completeness of case capture) and its specificity (distinguishing chikungunya from other cocirculating, syndromically similar arboviral diseases, such as dengue) are of paramount importance. Increased testing of suspected arboviral cases using low-sample-volume multiplex molecular assays has the potential to transform the diagnosis of chikungunya and other arboviral diseases. For example, a multiplex reverse transcription polymerase chain reaction assay that distinguishes between chikungunya, dengue, and Zika viruses is currently available, and it is being gradually adopted by laboratories worldwide. However, wider uptake in resource-poor regions remains necessary. Using such assays would enable differential laboratory confirmation of patients with signs and symptoms consistent with dengue or arboviral infections. Another recommended approach is using a structured method of laboratory confirmation, which can improve risk estimates, even if some samples are not tested. In this scenario, a standardized sample of suspected arbovirus cases would be tested; it could be a random sample of all suspected arbovirus cases or include all suspected chikungunya cases and a predetermined number (e.g., every 10th) of suspected dengue cases. This method can also randomize selection by age groups or other informative covariates. This approach would be most useful in areas lacking resources to test every suspected arboviral case and still provides vital information to improve underlying burden and risk estimates.
- Implementing population-based serosurveillance in endemic countries and priority destinations. Sampling an age-structured representative population and assessing chikungunya antibodies (serosurveillance) would demonstrate the historical disease burden in a particular location. These data can be integrated with routine surveillance data and models to estimate the changing epidemiology of a specific region or population (e.g., pregnant women or immunocompromised persons), thereby improving specificities of risk assessment.
- Conducting molecular epidemiology. Viral genomic information can provide crucial insights into mosquito vector competence (e.g., detecting viral lineages associated with enhanced transmission by *Ae. albopictus*) and the virus’s regional movement through travelers and immigrants or the transport of infected mosquitoes using phylogeographic methods. Regularly sequencing viruses from diverse locations and traveler populations can provide critical insights into the dynamics of viral spread. This approach is still in its early stages, but it is becoming increasingly common. Making chikungunya virus genomes available during outbreaks for near-real-time risk assessments can be challenging, but integrating this molecular epidemiological information can refine risk assessments for future chikungunya viral spread and mutations that may increase transmission efficiency or alter geographic risk.
- Standardizing and improving the transparency of case definitions and surveillance approaches. Case definitions should be more transparent and standardized across reporting organizations. Systematic and detailed information about chikungunya and arboviral surveillance across different countries and health care systems should be available to offer insight into local health care and response contexts.
- Providing continuing education about chikungunya. Health care and public health professionals should receive ongoing education about chikungunya to maintain disease vigilance. Educational materials should emphasize that chikungunya rarely disappears completely from a region that has experienced an outbreak; rather, it is often underdiagnosed or misdiagnosed, particularly in endemic countries that lack the molecular or serological testing capacity for accurate diagnosis.
- Enhancing data availability, quality, and connectivity. Outbreak-reporting resources should provide more comprehensive demographic data (e.g., age) to support accurate burden and risk models and assessments. Building connections among diverse data sources and developing methodologies to address variations in reporting and quality constitute steps needed to optimize use of existing data sources.
- Using traveler surveillance data to estimate traveler incidence rates. Although not commonly available, traveler incidence rates are vital to understanding the landscape of traveler risk. Individual cases or case series of chikungunya in travelers have been used to identify an elevated risk of chikungunya and to demonstrate chikungunya virus transmission in places without local reporting. For example, as travelers return to higher-resource settings and seek care for chikungunya, their health care provider reports the case, which can serve as a sentinel indicator of an outbreak.^32^^,^^33^ Sometimes, this scenario may occur sooner than a country reports an outbreak itself.^25^^,^^26^ This approach can serve as a proxy for chikungunya surveillance in countries that lack surveillance or the ability to consistently and adequately report certain diseases.
- Combining and integrating existing and emerging data resources. Connecting and integrating various types of data are beneficial to all scientists, including modelers, clinicians, and virologists. Currently, various environmental risk maps and local outbreak-reporting data typically exist in isolation. High-quality traveler surveillance data could be combined with timely sources of local case data to better predict outbreak trajectories and spread, leading to improved applications of preventive care and clinical decision-making. One source of information is rarely adequate for covering all clinical needs as most current systems contain at least one significant weakness in terms of quality, speed, and trust. Existing modeling approaches can already integrate multiple data sources when publicly available, and the continued development of artificial intelligence-driven resources may provide additional opportunities to address this concern. Additionally, combining surveillance data with viral genomic information could reveal clinically relevant genetic modifications in circulating strains.

### Recommendations to improve chikungunya risk communication to travelers.

- Raise awareness of chikungunya as a travel health concern. Many routine travelers, health care providers, and medical officers within international organizations are often unaware of chikungunya, its risks, and its health consequences. Even with better outbreak surveillance and reporting, stakeholders connected to the tourism industry may not encourage such visibility. Several strategies to enhance visibility among travelers include 1) ensuring the dissemination of current and accurate chikungunya information to publicly accessible sites frequented by travelers, particularly those that provide pretravel consultation and vaccination services; 2) offering web-based and other virtual resources for clinicians to learn about chikungunya and prevention strategies (e.g., sponsored webinars); and 3) disseminating data at pertinent medical conferences (e.g., those focused on tropical medicine and traveler medicine).
- Clearly articulate the possible severe health repercussions. Heightened traveler awareness should include chikungunya’s severe sequelae. Emphasizing the high frequency of arthralgia as well as the relatively rare neurological and cardiac complications^34^ and elevated risk of perinatal complications^35^ is key. This information may underscore the importance of prophylactic actions during travel and pretravel vaccination, especially for high-risk groups.
- Ensure that traveler educational materials are easily interpretable, timely, and transparent concerning the origin of the information and that they accurately represent changes in risk levels. At times, traveler risk websites lack transparency regarding the sources of the posted information and their risk alerts. Showing a clear pathway that illustrates the risk alert’s origins can foster trust and increase the use of that resource by travelers. Also, some risk alerts are promptly removed not because of the absence of disease exposure risk but rather, because of the diminished novelty of that risk. Maintaining alerts on websites for the duration of heightened transmission ensures that travelers are adequately informed about the exposure risk associated with their destination.

## Supplemental Materials

10.4269/ajtmh.26-0117 Supplemental Materials
