# Supplementary material for: Expert Recommendations to Strengthen Chikungunya Outbreak Surveillance and Reporting for Traveler Health Protection
Source: Am J Trop Med Hyg. 2026 May 26;115(1):121–9. doi: 10.4269/ajtmh.26-0117 (PMC13326870; doi:10.4269/ajtmh.26-0117)
Supplement: Supplemental Materials [file tpmd260117.SD1.pdf]

Supplemental materials for *Expert recommendations to strengthen chikungunya outbreak surveillance and reporting for traveler health protection*

Table S1. Expert Panel Discussion Questions

#	**Question**
1	In the recent pre-session informational survey, you identified several resources for obtaining information about a chikungunya outbreak. What are the strengths of these resources?
2	Are there particular aspects of your chosen resources that are not ideal? Are there hurdles or issues you must overcome to get the data you need for informed decision-making?
3	Have your trusted or go-to reporting sources changed in the last year (2025)?
4	Is there an ideal combination of resources for assessing chikungunya risk? Is there an easy way to cross-validate across those trusted sources?
5	What would you recommend as additional strategies to improve chikungunya surveillance?

Supplemental Figure S1: Comparison of reported data types across the outbreak reporting resources.

Resources	Confirmed Cases	Outbreak Reports & Alerts	Travel Advisories & Risk Assessments	Epidemiological Trends & Burden Estimates	Research Data (molecular, surveillance, case reports)	Narrative Reports / Descriptive Data	Early Warning / Biothreat Alerts	Vaccine Recommendations
BEACON		●					●	
HealthMap		●						
WHO	●	●	●					
GIDEON	●	●		●				
Shoreland Travax		●	●					●
ProMED		●				●	●	
Africa CDC	●	●	●	●				
ECDC	●	●		●				
PAHO	●	●		●				
US CDC	●	●	●	●				●
MoH reports	●	●		●				●
Institut Pasteur		●		●	●			
GeoSentinel Network	●				●			
NGO reports		●				●		
Peer-reviewed publications	Varies	●		Varies	●	●		
Media		●				●		

Supplementary Results

1. Comparison of chikungunya outbreak reporting and risk assessment tools

Most chikungunya outbreak reporting and risk assessment resources provide multiple types of outbreak-related information (Figure S1). The most commonly reported features across all the resources were outbreak reports and alerts (15/16), followed by reporting confirmed cases (8/16). The sources most likely to report confirmed case counts were various international, regional, or national governmental entities (e.g., WHO, national or regional CDCs, and MoHs). The GeoSentinel Surveillance Network and GIDEON also report confirmed cases and case counts. Four resources specifically report travel advisories or risk assessments: the WHO, US CDC, Africa CDC, and GIDEON. Three others provide vaccine recommendations (US CDC, Shoreland Travax, and MoH reports).

2. Descriptions of surveillance and outbreak reporting resources and relevant expert panel remarks

a.) European Centre for Disease Prevention and Control (ECDC)

ECDC, the EU's public health agency, gathers and shares data on infectious diseases, evaluates risks to European populations, and advises member countries on preventing and responding to outbreaks. The ECDC website posts weekly bulletins on active health threats (*Communicable Disease Threats Reports [CDTR]* [1]) to the EU, providing information on local transmission, importation of cases, and seasonal trends of infectious diseases of concern. ECDC supports multiple surveillance networks in the EU, gathering data from several laboratories and monitoring sites. It also gathers data from international (WHO) and regional sources (PAHO) as needed to describe outbreaks in other countries. ECDC specifically includes data on the potential origin of travel-related cases (e.g., the country visited by the traveler where the infection was acquired), which the expert panel noted as particularly useful. It also includes detailed lists of each outbreak cluster, specifying location, case counts, and other key features as emphasized by the expert panel. The detailed metadata provided by the ECDC is seldom available in arbovirus surveillance in either destination or endemic countries. The panelists universally emphasized that ECDC is a particularly strong resource for chikungunya reporting. The panel recognized it as one of the top two resources for reporting lab-confirmed cases, alongside US CDC.

b.) World Health Organization (WHO)

The WHO is an international health agency that promotes global health by guiding and coordinating international health initiatives and responses to health emergencies, establishing health standards, preventing disease, and offering technical assistance. The WHO updates its online reporting weekly for diseases of immediate concern and situationally for others not involved in active outbreaks. The *Weekly Epidemiological Record* [2] offers a summary of epidemiological information regarding communicable disease outbreaks of public health

importance; however, this resource was noted for its slow dissemination. The WHO disseminates information regarding confirmed cases, outbreak activities, and risk assessments. It collects data from multiple sources but primarily relies on standardized reporting systems that convey information from the national surveillance systems of its 194 member nations. The panelists recognized its extensive global coverage and data structure as its primary strengths.

d.) Pan American Health Organization (PAHO)

As the WHO's Regional Office for the Americas, PAHO aims to strengthen health systems, respond to health emergencies, and improve the equity and sustainability of national and local health systems in the Americas. PAHO offers regularly updated *Epidemiological Alerts and Updates* [3] as public resources. These reports provide information on global health events relevant to the Western Hemisphere, often issued initially as alerts that are subsequently updated to reflect situational developments. PAHO compiles and disseminates these data from official national sources (such as MoHs) within member states, regional surveys, and the Arbovirus Diagnostic Laboratory Network of the Americas [4]. PAHO also incorporates data from WHO collaborating centers, national websites, civilian and military laboratories, and nongovernmental organizations. The panelists affirmed that PAHO serves as an excellent resource for chikungunya reporting in the Americas, citing its frequent dissemination of accurate and regionally pertinent data.

e.) Peer-reviewed publications

Most panelists identified peer-reviewed publications as frequently consulted and trusted sources for in-depth information; however, they typically combined them with more timely reporting resources. Panelists noted that while peer-reviewed publications may not be useful for real-time risk assessments, they offer essential context and methodological depth to elucidate site-specific

outbreak dynamics and emerging trends. The types of information provided by peer-reviewed sources, such as genomic and seroprevalence data [5,6] are critical to surveillance and understanding outbreak dynamics, but often are not included in data aggregators or international resources. These insights can enhance the overall understanding of chikungunya outbreaks, particularly for modeling, and inform the evaluation of preventive tools by offering retrospective context from past outbreaks.

f.) United States Centers for Disease Control and Prevention (US CDC)

The US CDC, the US national health agency, focuses on detecting and responding to health emergencies, preventing diseases and injuries, and promoting health equity. The US CDC's publicly available resources are updated at variable intervals; however, delays in reporting can be significant. The US CDC typically conducts robust arboviral surveillance through ArboNet [7] and disseminates the resulting laboratory-confirmed, passive surveillance findings on its website following data cleaning and analysis. Despite high data quality, these surveillance data rarely provide real-time updates on arboviral outbreaks, which are vital for clinician panelists. Travel Health Notices (THN; [8]) include public alerts regarding disease outbreaks and sporadic disease cases in unusual or new geographic locations. These alerts are issued following consultation with trusted local sources and US CDC subject matter experts. The panelists identified reporting of laboratory-confirmed cases as a particular strength of the US CDC. While at the time of the expert panel meeting the US CDC had recently expanded the list of countries for which it reports chikungunya outbreak activity, panelists noted that its updates are not always sufficiently focused or timely.

g.) National/local governmental resources

Most countries have national public health agencies (e.g., MoHs) and often conduct local surveillance activities (e.g., regional, state, or city-level monitoring). These entities are the foundation of most international disease reporting resources and higher-level data aggregators. Therefore, all users either directly or indirectly rely on these national or local reporting mechanisms. Despite their importance, these entities often have suboptimal resources for conducting surveillance and outbreak reporting. In some countries, publicly available annual reports are published on a fixed schedule, not necessarily in temporal alignment with an ongoing outbreak, even if chikungunya is a notifiable disease.

h.) ProMED, Shoreland Travax, and GIDEON

The expert panel highlighted three additional resources that they also consult for risk assessment—ProMED, an open-source reporting system with expert vetting and interpretation; Shoreland Travax, an online clinical decision support tool; and GIDEON, a commercial surveillance database. In contrast to the previously listed resources, which were all publicly available, Shoreland Travax, GIDEON, and ProMED are entirely subscription-based. All three of these resources are typically consulted in combination with the trusted resources listed above and offer timely, complementary information in user-friendly formats.

Data aggregators provide the timely and accurate information needed to inform immediate decisions on clinical care and vaccinations. Independently reviewing sources from the WHO, ECDC, and PAHO would be impractical and time-consuming in this context. Recently, the ACIP advised US travel medicine clinicians to rely on US CDC chikungunya outbreak data for such clinical recommendations [9]; however, that data is subject to surveillance delays, and agency disruptions have recently impacted the timeliness and accuracy of the available data.

Shoreland Travax promptly updates and processes outbreak information, integrating it with vaccine recommendations to make it an efficient resource for travel clinicians. GIDEON was described as a unique resource because it provides case counts, treatment courses, and testing data (where available). GIDEON often presents the outbreak's epidemiological curve (a histogram of cases over time illustrating the outbreak's trajectory) alongside trend indicators indicating whether the outbreak's magnitude is increasing or decreasing, a feature uncommon across other resources.

i.) Emerging resources

BEACON is a new, open-access resource that aims to provide carefully vetted information on the early detection of global disease threats through rapid reporting and open sharing of information. Although the panelists were largely unfamiliar with BEACON, this platform is an example of AI-driven interfaces that aggregate and report outbreak data in real time. It was identified as being at an early stage of development at the time of the panel but may represent a future direction of outbreak reporting.

Another unleveraged resource identified by the panelists was the Arboviruses Action Committee [10], part of the Global Virus Network. This informal committee consists of subject matter experts who meet bi-monthly and share their observations and assessments of arbovirus activity in their host country and neighboring countries. Frequently, this information is more detailed than what is provided by more formal reporting mechanisms described above; however, it primarily reflects the committee's geographic coverage. While not currently public, identifying ways to disseminate this information can help strengthen existing chikungunya outbreak reporting pipelines.

References

1. ECDC., 2022. Weekly threats reports (CDTR). Available at: https://www.ecdc.europa.eu/en/publications-and-data/monitoring/weekly-threats-reports. Accessed. May 22, 2022
2. WHO. The Weekly Epidemiological Record (WER). Available at: https://www.who.int/publications/journals/weekly-epidemiological-record/. Accessed
3. PAHO. Epidemiological alerts and updates - PAHO/WHO | Pan American Health Organization. Available at: https://www.paho.org/en/epidemiological-alerts-and-updates. Accessed
4. PAHO. The Arbovirus Diagnosis Laboratory Network of the Americas (RELDA) - PAHO/WHO | Pan American Health Organization. Available at: https://www.paho.org/en/topics/dengue/arbovirus-diagnosis-laboratory-network-americas-relda. Accessed
5. Parker DM, Haileselassie W, Hailemariam TS, Workenh A, Workineh S, Wang X, Lee M-C, Yan G., 2025. High seroprevalence of antibodies to Dengue, Chikungunya, and Zika viruses in Dire Dawa, Ethiopia: A cross-sectional survey in 2024. *PLoS Negl Trop Dis 19*: e0013357
6. Khongwichit S, Chuchaona W, Korkong S, Wongsrisang L, Thongmee T, Poovorawan Y., 2025. Chikungunya virus in Thailand (2020–2023): Epidemiology, clinical features, and genomic insights. *PLoS Negl Trop Dis 19*: e0013548
7. CDC., 2024. ArboNET. Available at: https://www.cdc.gov/mosquitoes/php/arbonet/index.html. Accessed. April 20, 2024
8. CDC. Travel Health Notices | Travelers' Health | CDC. Available at: https://wwwnc.cdc.gov/travel/notices. Accessed
9. CDC., 2025. ACIP Presentation Slides: April 15-16, 2025 Meeting. Available at: https://www.cdc.gov/acip/meetings/presentation-slides-april-15-16-2025.html. Accessed. April 16, 2025
10. Global Virus Network., 2023. Action Committees. Available at: https://gvn.org/activities/collaborative-groups/. Accessed. August 17, 2023
